# Comparison of Two Blood-Based Genotyping Tests to Investigate the KRAS G12C Mutation in Patients with Non-Small-Cell Lung Cancer at Failure of First-Line Treatments

**DOI:** 10.3390/diagnostics11122196

**Published:** 2021-11-25

**Authors:** Chiara Nicolazzo, Alain Gelibter, Irene Bottillo, Francesca Belardinilli, Simona Pisegna, Gianluigi De Renzi, Daniele Marinelli, Paola Grammatico, Enrico Cortesi, Giuseppe Giannini, Paola Gazzaniga

**Affiliations:** 1 Cancer Liquid Biopsy Unit, Department of Molecular Medicine, Sapienza University of Rome, 00161 Rome, Italy; gianluigi.derenzi@uniroma1.it (G.D.R.); paola.gazzaniga@uniroma1.it (P.G.); 2 Department of Radiology, Oncology and Pathology, Sapienza University of Rome, 00161 Rome, Italy; alain.gelibter@uniroma1.it (A.G.); simona.pisegna@uniroma1.it (S.P.); daniele.marinelli@uniroma1.it (D.M.); enrico.cortesi@uniroma1.it (E.C.); 3 Laboratory of Medical Genetics, Department of Molecular Medicine, Sapienza University, San Camillo-Forlanini Hospital, 00152 Rome, Italy; irene.bottillo@uniroma1.it (I.B.); paola.grammatico@uniroma1.it (P.G.); 4 Laboratory of Molecular Oncology, Department of Molecular Medicine, Sapienza University of Rome, 00161 Rome, Italy; francesca.belardinilli@uniroma1.it (F.B.); giuseppe.giannini@uniroma1.it (G.G.)

**Keywords:** KRAS G12C, liquid biopsy, clonal evolution, non-small-cell lung cancer, circulating tumor DNA

## Abstract

Although molecular profiling at diagnosis has traditionally relied on direct sampling of neoplastic tissue, cancer clonal evolution represents a critical obstacle to use primary tissue biopsies to guide clinical decision-making at the time of progressive disease. Liquid biopsies might offer enormous advantages over tissue biopsies, tracking in real-time temporal-based tumor dynamics following each line of treatment. Here, we compared two liquid biopsy assays, specifically real-time polymerase chain reaction and next-generation sequencing, to track the KRAS G12C mutation at onset of progression from previous lines of therapy. The KRAS G12C mutation was acquired at the time of progressive disease in 24% of patients. Furthermore, all patients with KRAS G12C mutation-positive tissue became negative in ctDNA at progressive disease. The presence of other somatic mutations in all these samples confirmed the tumor origin of the circulating DNA. This pilot study suggests that in the assessment of the plasma KRAS G12C mutation as a druggable target, real-time PCR assay Idylla might be a suitable approach to better match patients to interventional biomarker-targeted therapies.

## 1. Introduction

The rapid evolution of precision medicine in non-small-cell lung cancer (NSCLC) led to an increased demand for biomarkers in a very short timeframe, with molecular testing being mandatory to assign patients to specific treatment groups in precision oncology trials [1]. Approximately 30% of patients with NSCLC have RAS mutations, with 13% having the KRAS G12C mutation, a single point mutation with a glycine-to-cysteine substitution at codon 12 [2]. AMG-510 (sotorasib) is a small molecule that selectively and irreversibly targets KRAS G12C through a unique interaction with the P2 pocket, irreversibly locking KRAS in its inactive GDP-bound state [3]. Based on the results of the phase 2 CodeBreaK 100 trial, a new drug application was recently submitted to FDA for sotorasib for the treatment of patients with KRAS G12C-mutated locally advanced or metastatic NSCLC after at least one previous systemic therapy attempt [4]. A phase 3 study comparing sotorasib with docetaxel in NSCLC patients is currently ongoing [5]. The trial will recruit patients with pathologically documented, previously treated NSCLC with evidence of the KRAS G12C mutation in the archived primary tumor tissue as confirmed through molecular testing. Although molecular profiling at diagnosis has traditionally relied on direct sampling of neoplastic tissue, cancer clonal evolution represents a critical obstacle to use primary tissue biopsies to guide clinical decision-making at the time of progressive disease. In fact, primary tissue analysis in patients who need to be matched to interventional biomarker-targeted therapies at the time of progression is often not informative as the archival tissue collected at a single timepoint may not account for spatial and temporal cancer heterogeneity. Thus, the genomic profile of a tumor at the time of diagnosis might significantly differ from that observed at progressive disease [6]. In this respect, liquid biopsies might offer enormous advantages over tissue biopsies, mainly to track in real time temporal-based tumor dynamics following each line of treatment. In this proof-of-concept study, we sought to investigate the utility of two different blood-based KRAS G12C genotyping tests to be performed at the time of progressive disease (PD) to better match patients to interventional biomarker-targeted therapies. For this purpose, we compared pretreatment biopsy samples and plasma circulating tumor DNA (ctDNA) at the time of PD for the KRAS G12C mutation.

## 2. Materials and Methods

### 2.1. Study Population

Plasma samples were collected from thirty-eight NSCLC patients at the time of progression on any first-line treatment (platinum-based doublet chemotherapy/checkpoint inhibitors/targeted therapy). All the patients were treated at our department between January 2018 and February 2020 at Policlinico Umberto I of Rome. The inclusion criteria were as follows: males or females aged >18 years; mutational analysis available on the primary tumor tissue from the targeted gene panels; one previous chemotherapy regimen received; documented progressive disease confirmed by imaging; measurable disease according to the response evaluation criteria in solid tumors (RECIST) criteria, v.1.1; ECOG performance status ≤2; signed informed consent. All the plasma samples were obtained at the time of PD from the first treatment line. Authorization to perform liquid biopsies was released by the Regional Ethical Committee (No. 179/16), and the study was conducted in accordance with the Declaration of Helsinki.

### 2.2. Targeted Sequencing of the Pretreatment Tumor Samples

The mutational analysis of the primary tumor tissue was performed as part of the routine diagnostic process using the Ion Torrent Personal Genome Machine (IT-PGM). IT-PGM sequencing was achieved as described [7]. The tissue samples were analyzed using Ion AmpliSeq Colon and Lung Cancer Research Panel V2 (CLV2, Thermo Fisher Scientific, Guilford, CT, USA) containing a single primer pool to amplify hotspots and targeted regions of 22 cancer genes frequently mutated in colorectal cancers and NSCLCs.

### 2.3. Analysis of the ctDNA Samples at the Time of Disease Progression

Blood samples (10 mL) prospectively obtained for ctDNA analysis at the time of PD were collected in K_2_EDTA tubes and immediately processed. The blood samples were centrifuged at 1500 rpm for 10 min; then, plasma was removed and further centrifuged at 13,000 rpm for 1 min. The plasma samples were aliquoted. One aliquot (1 mL) was used to screen for the KRAS G12C mutation through the real-time polymerase chain reaction (PCR) (Idylla^TM^, Biocartis) Jersey City, NJ, USA) according to the manufacturer’s instructions. The plasma samples were further processed for IT-PGM sequencing. For this purpose, ctDNA was purified from plasma for each patient (4 mL) with a Maxwell 16 system (Promega) using a Maxwell RSC ccfDNA plasma kit according to the manufacturer’s instructions. Eluted ctDNA quantity was assessed with a Qubit 2.0 Fluorometer (Thermo Fisher Scientific) using a Qubit™ dsDNA HS assay kit (Thermo Fisher Scientific). Cell-free (cf) DNA and cfRNA (i.e., cfTNA, cell-free total nucleic acid) were purified from plasma for each patient (2 mL) with a MagMAX™ cfTNA Isolation Kit (Thermo Fisher Scientific) according to the manufacturer’s instructions. Eluted cfDNA quantity was assessed with the Qubit 2.0 Fluorometer using the Qubit™ dsDNA HS assay kit; the cfTNA samples were analyzed using the Oncomine Lung Cell-Free Total Nucleic Acid Research Assay (Thermo Fisher Scientific) that allows the identification of hotspots single nucleotide variants (SNVs) and short indels in 11 genes (i.e., ALK, BRAF, EGFR, ERBB2, KRAS, MAP2K1, MET, NRAS, PIK3CA, ROS1, TP53), gene fusions affecting ALK, RET, and ROS1 genes, and of MET copy number variants (CNVs) and exon 14 skipping. By using the tag sequencing technology, a limit of detection (LOD) of 0.1% can be achieved. Briefly, 20 ng of cfTNA input or a maximum volume of 13 µL per sample were used for libraries preparation, according to the manufacturer’s instructions. Templated spheres were prepared using 100 pM of each library using an Ion Chef machine (Thermo Fisher Scientific). Template-positive spheres were loaded into Ion Chip 530 and sequenced using an IT-S5Xl machine (Thermo Fisher Scientific). The sequencing data were analyzed with the Ion Torrent Suite Software (Thermo Fisher Scientific, http://github.com/iontorrent/TS, accessed on 24 November 2021) and the Ion Reporter Software according to the company’s recommendations. The variants were verified using the IGV visualization tool (http://www.broadinstitute.org/igv/, accessed on 24 November 2021).

## 3. Results

Thirty-eight patients with advanced NSCLC were prospectively enrolled at the time of PD after the first-line treatment. The archived tissue biopsy samples had been previously processed using Ion AmpliSeq Colon and Lung Cancer Research Panel V2 (CLV2, Thermo Fisher Scientific, Guilford, CT, USA). Nineteen patients were enrolled at failure of the first-line chemotherapy (CT) regimen, while 13 and six—at failure of the first-line immunotherapy (IT) and targeted therapy (TT), respectively.

### 3.1. Mutational Analysis in the Pretreatment Tissue Samples

In the primary tumor biopsies, KRAS was found mutated in 13/38 cases (34%). The most frequently detected mutation was G12C (47%), followed by G12D (23%), G12F (15%), and Q61 (15%). Therefore, in the whole patient population, the KRAS G12C mutation was detected in 16% of the patients at the time of first diagnosis. Among the six patients with the KRAS G12C mutation, one had a co-occurring CTNBB1 mutation, while five had no additional comutations detected (Table 1).

### 3.2. KRAS G12C Mutation in Plasma ctDNA at Disease Progression

To examine changes in the KRAS G12C mutational status occurring during treatment, ctDNA analyses were performed on the blood samples prospectively obtained from the patients with radiologically confirmed disease progression. All the plasma samples obtained at PD were first screened for the KRAS G12C mutation through the Idylla test. The KRAS G12C mutation was detected in nine out of the 38 ctDNA samples (24%). In all these cases, the KRAS G12C mutation emerged at PD, although was not previously detected in the primary tumor tissue.

Analysis of the plasma samples through NGS confirmed the results obtained through Idylla in 100% of the cases. KRAS G12C coexisted with EGFR mutations in two cases; MAP2K1 in two cases; p53 in two cases; BRAF in one case; PIK3A in one case. G12C co-occurred with other KRAS mutations in one case. All the patients with the baseline tissue samples positive for the KRAS G12C mutation became negative in ctDNA at PD. The presence of other somatic mutations in all these samples allowed excluding that the lack of the KRAS G12C mutation detection in plasma at PD might be due to the scarce release of ctDNA (Table 1).

## 4. Discussion

The success of targeted therapies being closely dependent on the identification of the target, the allocation to precision medicine trials is usually based on DNA sequencing of the primary tumor biopsy. The clonal evolution of tumors defined by changes in the mutational profile between diagnosis and relapse is conceivably the most significant barrier in the treatment of patients with targeted therapies. By changing their mutational profile, tumor cell populations adapt to the new environment imposed by treatments, in turn shaping malignant progression. For this reason, cancer evolution during the first and subsequent lines of therapy render archival samples not representative of the genomic profile of cancer at the time of PD. In fact, mutations as therapeutic targets originally detected in the primary tumor tissue might not be present at the time of relapse or, alternatively, might develop at the time of PD, posing a clinical challenge in regards to rebiopsy at the time of PD. This goal, although desirable, is unfeasible or too demanding for most lung cancer patients in view of the risk of complications from invasive manipulations. In this respect, liquid biopsy performed at the time of PD may provide real-time information to disease changes and better match patients to subsequent interventional biomarker-targeted therapies. Recently, KRAS testing in advanced-stage NSCLC patients has acquired a novel predictive significance, the KRAS G12C mutation being target of different small molecules currently being tested in clinical trials involving patients with advanced NSCLC in whom the actionable mutation is assessed in archived tumor tissues [8]. Recent evidence has been provided that KRAS mutation assessment in NSCLC baseline plasma samples is feasible, especially in patients in whom tumor tissue is not available for molecular testing [9]. Several commercial liquid biopsy platforms are available, ranging from PCR-based analysis to broad targeted NGS applications. Here, we compared two liquid biopsy assays to track the KRAS G12C mutation at onset of progression from previous lines of therapy. The fully automated Idylla device which we used to screen for the KRAS G12C mutation has several advantages, including the fast workflow without the need for preanalytical DNA extraction, the low cost, and the ease of execution for molecular genotyping. The analytical sensitivity of Idylla in detecting KRAS mutations has been reported to be in the range of 0.1–1% and a recent comparison between Idylla and NGS for plasma KRAS mutations in NSCLC showed a very high overall agreement [10]. On the other hand, especially in NSCLC, NGS has the main advantage of analyzing several genes in parallel with resolution down to 0.1% allele frequency, despite its complex workflow and a turnaround time of several days compared to Idylla. Since in our series the two methods gave concordant results in 100% of the cases, we suggest that in the specific assessment of the plasma KRAS G12C mutation as a druggable target, Idylla might be more cost-effective in routine clinical practice than NGS. On the other hand, in all the plasma samples with wild-type KRAS G12C mutation at progression, the detection through NGS of other somatic mutations allowed us to confirm the presence of a sufficient amount of ctDNA.

The high percentage of conversions from a KRAS mutant in primary tissues to KRAS wt in plasma at PD and vice versa reported in our series deserves a further comment. Liquid biopsy-guided genomic studies performed in colorectal cancer have demonstrated a significant increase in RAS mutant clones in plasma at the onset of secondary resistance to anti-EGFR therapy [11]. Similarly, the disappearance of RAS mutant clones in plasma has been demonstrated in a high percentage of patients who failed first-line treatments [12]. This frequent modulation of RAS mutations at the time of progressive disease had previously been described in leukemia patients as well [13].

To our knowledge, this is the first proof-of-concept prospective study aimed to evaluate the utility of two different blood-based KRAS G12C genotyping tests to be performed at the time of progressive disease to better match patients to interventional biomarker-targeted therapies. Our results underline the risk of restricting biomarker studies to the analysis of the primary tumor tissue for clinical trial stratification of cancer patients at the time of progressive disease, particularly if the tissue biopsy used for biomarker evaluation had been performed long before disease progression. Whenever a biopsy on progression is not feasible, liquid biopsy might fill the gap due to its lack of invasiveness, easy accessibility, and good reproducibility.

Our study has several limitations, including the small sample size. First, since the patients were enrolled at the time of PD, we could not match the KRAS G12C status in the tumor tissues and plasma samples at the time of diagnosis. In this regard, the unavailability of ctDNA samples at baseline could represent a bias in the interpretation of our results. In fact, if it is widely accepted that tissue biopsy may not take into account the constitutive heterogeneity of the tumor, liquid biopsy has several limitations as well, that need to be considered in the interpretation of data [14,15]. First, the scarce release of ctDNA in patients with brain metastases poses a significant challenge for the sensitivity of plasma-based assays in some clinical contexts. Furthermore, most plasma ctDNA assays do not use matched sequencing of white blood cells; thus, false-positive results can occur due to clonal hematopoiesis as well as sequencing artifacts. False-positive results might also depend on the presence of variants derived from synchronous primary cancers. A further limitation of this study is that NGS panels for blood and tissue profiling were not matched for sequenced genes, preventing us from making further assumptions on mutational co-occurrences in our cohort. Finally, the too small number of patients enrolled did not allow us to establish whether the modulation of the KRAS G12C mutation might be associated with a particular first-line treatment. To answer this question, a prospective study with a larger number of patients would be needed. In order to prove that the treatment guidance based on the mutational status of the liquid biopsy at the time of progression will be more beneficial for patients compared to that based on the mutational status of the archived tumor biopsy at the time of diagnosis, a randomized prospective study would be desirable.

## Figures and Tables

**Table 1:** Mutational analysis in the pretreatment tissue samples and in plasma ctDNA at disease progression.

Patients Characteristics				Tissue Molecular Analysis (Baseline)					Plasma Molecular Analysis (PD)		
Pt.#	Age	Gender	Smoking Status	ALK	ROS	PD-L1	KRAS	Other	KRAS (Idylla)	KRAS (NGS)	Other
1	71	M	2	0	0	1	wt	DDR2	G12C+	G12C+	MAP2K1
2	56	M	1	0	0	0	G12C−	CTNNB1	wt	wt	BRAFV600E
3	65	M	2	0	0	0	wt	None	wt	wt	none
4	73	M	2	0	0	0	wt	None	G12C+	G12C+	PI3K
5	65	M	1	0	0	1	wt	P53	wt	wt	none
6	64	F	1	0	0	1	wt	P53	G12V	G12V	none
7	70	M	1	0	0	0	wt	P53	G12C+	G12C+	MAP2K1
8	77	F	2	0	0	1	G12C−	P53	wt	wt	P53
9	76	M	2	0	0	0	G12F	p53	wt	wt	none
10	66	M	1	0	0	1	wt	p53; STK11	G12C+	G12C+	EGFR
11	65	M	1	0	0	1	wt	P53	wt	wt	none
12	64	F	1	0	0	1	wt	P53	wt	wt	none
13	72	M	2	0	0	0	wt	p53; MET	wt	wt	EGFR
14	58	F	2	0	0	1	Q61H	none	wt	wt	none
15	72	F	2	0	0	1	wt	none	wt	wt	none
16	67	F	2	0	0	1	Q61K	none	wt	wt	none
17	77	F	2	0	0	0	wt	AKT	wt	wt	EGFR del 19
18	84	M	1	0	0	0	G12D	STK11	G12D	G12D	none
19	71	M	1	0	0	1	wt	STK11; CTNNB1	wt	wt	
20	44	M	2	0	0	0	wt	none	wt	wt	none
21	81	M	2	0	0	1	G12F	p53; NRAS; ERBB4	G12C+	G12C+	P53
22	63	F	2	0	0	2	wt	P53	wt	wt	PIK3A
23	56	M	2	0	0	2	wt	P53	G12C+	G12C+	P53
24	76	M	2	0	0	2	G12C−	none	wt	wt	BRAF V600
25	78	M	1	0	0	2	G12D	none	G12D	G12D	BRAF V600E
26	65	F	1	0	0	2	G12D	P53	G12D	G12D	P53
27	60	M	1	0	0	2	wt	none	G12C+	G12C+	BRAFV600
28	65	F	0	0	0	2	wt	none	G12C+	G12C+	G12D
29	55	M	2	0	0	2	wt	none	wt	wt	MET
30	62	F	1	0	0	2	G12C−	none	wt	wt	PIK3A
31	69	M	2	0	0	2	G12C−	none	wt	wt	MAP2K1
32	58	M	2	0	0	2	G12C−	none	wt	wt	BRAFv600E
33	71	F	0	0	0	0	wt	EGFR; MET	wt	wt	T790M
34	60	M	0	0	0	2	wt	EGFR; p53	wt	wt	T790M
35	64	M	2	1	0	0	wt	p53; PIK3A	wt	wt	none
36	78	M	0	0	0	0	wt	EGFR; PIK3A	wt	wt	T790M
37	87	M	2	1	0	2	wt	none	G12C+	G12C+	EGFR
38	35	M	0	0	0	1	wt	EGFR	wt	wt	none

G12C−: patients with the baseline tissue samples positive for the KRAS G12C mutation who became negative in ctDNA at PD. G12C+: patients with the baseline tissue samples negative for the KRAS G12C mutation who acquired a mutation in ctDNA at PD. PD: progressive disease.

## Data Availability

The data presented in this study are available on request from the corresponding author.
